# Owner-Reported Pica in Domestic Cats Enrolled onto a Birth Cohort Study

**DOI:** 10.3390/ani11041101

**Published:** 2021-04-12

**Authors:** Rachel Kinsman, Rachel Casey, Jane Murray

**Affiliations:** Bristol Veterinary School, University of Bristol, Bristol BS40 5DU, UK; rachel.casey@dogstrust.org.uk (R.C.); jane.murray@dogstrust.org.uk (J.M.)

**Keywords:** domestic cat, pica, wool-sucking, behavioural disorders, questionnaire, longitudinal study

## Abstract

**Simple Summary:**

This study investigated the types of materials targeted by cats eating non-nutritive materials (‘pica’), at about 6, 12 and 18 months of age, as reported by owners. Pica was most common at about 6 months, as compared to the older age categories. Most cats targeted a single type of material, with plastics and other materials being chewed or eaten more commonly than wool or other fabrics. The factors associated with the occurrence of “chronic pica” (pica exhibited at all three timepoints) in cats were also investigated. Moving to a new house, renting rather than owning a home, and living in a household without a dog(s) were factors found to increase the odds of a cat displaying chronic pica.

**Abstract:**

The prevalence and cooccurrence of pica towards different target materials were investigated using prospective data from three questionnaires completed by owners participating in a longitudinal study of UK pet cats. Pica towards one or more material types was reported in 42.9% (229/534), 32.0% (171/534), and 30.9% (165/534) of cats aged approximately 6, 12, and 18 months, respectively. At all timepoints, it was most common for only one material type to be targeted. Associations between potential explanatory variables and “chronic pica” (pica exhibited at all three timepoints) were also explored. Multivariable logistic regression revealed moving to a new house when the cat was aged approximately 6–12 months, renting rather than owning a home, and living in a household without a dog(s) when the cat was aged 2–4 months increased the odds of chronic pica occurrence. This study provides novel data from a cohort of UK pet cats and it is hoped this will increase the understanding of pica and provide direction for areas for future research.

## 1. Introduction

The term “pica” is used to describe the ingestion of non-nutritive items. It is known that cats target a range of items including fabrics (made of wool, cotton, or synthetic materials), shoelaces, rubber, plastics, paper, cardboard, wood, and metals [1,2,3].

Bradshaw and others broadened the definition of pica in cats to include chewing and/or sucking of non-nutritive items [1]. Whether these three behaviours (ingestion, chewing and sucking) should be grouped together or examined separately is debatable. Some suggest that chewing and/or sucking are kitten or infantile behaviours that are only retained by some into adulthood, therefore, the motivations for the behaviour may be different to that of ingestion [4,5]. Additionally, Borns-Weil and others reported that the age of onset of ingesting, chewing, and sucking differed by material type [2]. In this study, the term pica will be used to describe chewing of non-nutritive items with or without ingesting.

The short and long-term impacts on the health and welfare of cats that exhibit pica are largely unknown and require further investigation. However, theorised significant impacts include gastrointestinal problems (such as intestinal obstructions), nutrition absorption problems or inbalances, a reduced intake of food, feline infectious peritonitis, pyruvate kinase deficiencies, and wear or damage to teeth and gums [4,6,7,8,9,10]. It should be noted that the definition of pica used by the majority of the authors of these studies is limited or not provided, and in some of the studies the number of cats with pica was very low (i.e., <5 cats). Welfare impacts are unknown.

Although pica may be unlikely to be a direct cause of relinquishment to shelters, it has the potential to affect the human–animal bond. Owners of cats exhibiting pica may need to be vigilant to try and prevent ingestion of non-nutritive items, primarily for the welfare of the cat, but also to prevent damage to household items. Also, it could be hypothesised that an owner preventing access to a pica-targeted item might unwittingly cause frustration or distress to the cat, impacting on welfare.

There is limited research exploring the prevalence of pica in cats and the factors influencing onset and occurrence. Bradshaw and others suggested that the onset of pica could happen at any point during the first 4 years of life [1]. They found that onset within their study population most commonly occurred between 2–4 months of age and also noted that onset frequently occurred in the first 2 months following rehoming. This led the authors to theorise that separation from the mother and siblings and/or introduction to an unfamiliar environment could be associated with onset, but also recognised several factors could confound this, so this could potentially be a correlational association rather than a causal one. Another previous study reported that age of weaning appeared to be associated with pica in Birman cats [2], but no evidence of an association has been reported yet for other breeds [2,3].

Bradshaw and others also reported that for some cats within their study population the onset of pica occurred between 6–18 months of age, and in these cases, onset could not be linked to rehoming, so the authors suggested that sexual maturity or territorial behaviour could be associated with onset [1]. However, no evidence of an association between pica and neuter status, or pica and the sex of the cat, were reported in two other previous studies [1,3]. Bamberger and Houpt reported more male cats (21/32) than female cats exhibited pica in their study [11], but due to a relatively small sample size and lack of control population, those findings may be less robust than the studies with larger sample sizes. To the author’s knowledge, no longitudinal data on pica in cats has been reported. Whether pica is predominantly exhibited by kittens and is subsequently more or less likely to be retained as cats age would be useful to explore to increase the understanding of this behaviour.

Several studies of pica have focused on Oriental breeds [1,2]; this is perhaps due to anecdotal and clinical experience that suggested an increased problem in these breeds [12,13], but little evidence of associations exists between breed and pica.

Factors such as boredom and lack of social interactions have been speculated to be contributing factors for pica, however, a previous study reported pica did not appear to be a result of a suboptimal environment [3]. This is an area, however, that requires considerable exploration.

We hypothesise that pica is likely to be influenced by multiple factors, some of which may occur months or years before the owner considers the behaviour to be a problem. Whilst cross-sectional studies can identify associations, they cannot establish causality. Studies using pre-existing data generally include little environmental information. A better approach to elucidate the relative influence of environmental factors on behavioural outcomes is to recruit a cohort of kittens before these problems occur and follow them through life. Therefore, to provide a better understanding of pica and factors associated with the behaviour, this study used data collected prospectively to:

1. summarise the prevalence and cooccurrence of pica towards different target materials in cats aged 6, 12, and 18 months as reported by their owners, and;
2. identify and quantify early-life risk factors for the occurrence of pica exhibited by cats at all three timepoints, which we term ‘chronic’ pica. Exploring pica, which has been retained over time and become maladaptive, allows differentiation from “normal” or transient kitten behaviour.

## 2. Materials and Methods

### 2.1. Study Design and Participants

Data for this study were collected prospectively as part of the ‘Bristol Cats Study’ (BCS)—a longitudinal study of pet cats within the United Kingdom (UK). Between May 2010 and December 2013 (inclusive), 2203 cat owners were recruited to the study using a variety of advertising methods [14]. To be included in the BCS, the participants were required to: (1) live in the UK, (2) be aged 18 years or more, and (3) own a kitten (or kittens) aged 8–16 weeks at the time of registration.

BCS participants were asked to complete self-administered questionnaires (either online or via postal paper copies) when their cat reached specific ages. The data for this analysis were obtained from the first four questionnaires, which were issued between May 2010 and April 2015 [14]. Questionnaire 1 (Q1) was issued to owners of cats aged 2–4 months, Questionnaire 2 (Q2) at 6.5–7 months, Questionnaire 3 (Q3) at 12.5–13 months, and Questionnaire 4 (Q4) at 18.5–19 months.

The questionnaires consisted of mostly “closed questions” with multiple-choice answers. Data collected included the demographics of the owner, characteristics of the cat, and information relating to the management of the cat. All data from respondents were anonymised prior to analysis. The study was approved by the University of Bristol ethics committee (Reference UIN/13/026).

### 2.2. Outcome Variables

To investigate the occurrence of chronic pica within the BCS cohort, in Q2–4, owners were asked whether, at the time of questionnaire completion, their cat chewed with or without ingestion each of the following four materials: woollen fabrics, other fabrics, plastics, or other materials (Table S1 in Supplementary Materials).

For the risk factor analysis for pica, the two outcome categories were defined as:

1. cats whose owners had reported chronic pica towards one or more of the material types (woollen fabrics, other fabrics, plastics, or other materials) at all three time points (Q2–4); and
2. cats whose owners had reported never observing pica towards any of the material types (woollen fabrics, other fabrics, plastics, or other materials) at all three time points (Q2–4).

Cats reported by their owners to have exhibited pica intermittently toward one or more of the material types at one or two of the time points (Q2–4) were excluded from analysis.

### 2.3. Potential Explanatory Variables

Summarised in Table 1 are variables assessed for association with chronic pica, and included breed, acquisition age, sex, neuter status, appetite, and outdoor access. To enhance the statistical power of the analysis, especially when variables had categories that contained few data points, univariable analysis was utilized to justify combining categories that had similar associations with the outcome and where merging categories was judged to be logical.

### 2.4. Study Size

The study size was determined by the number of cats within the BCS study whose owners had completed Q1–4 (inclusive) and had completely answered the questions on pica (see Table S1 in Supplementary Materials) in Q2–4. If owners had responded “do not know” to the pica questions, they were excluded from analysis as they could not be placed into either of the outcome categories. To remove any effects of clustering at the level of the household, if an owner had registered more than one cat onto the BCS, one of their cats was randomly selected for inclusion in this analysis using a random number generator, and their other cat (or cats) were excluded. The study had 80% power to detect an odds ratio of ≥3 with a 95% confidence level assuming 50% of controls were exposed to the variables of interest (Epi-Info 7, CDC, www.epitools.ausvet.com.au/ -accessed on 30 June 2020).

### 2.5. Descriptive Statistics

The prevalence and co-occurrence of pica towards the four different materials (woollen fabrics, other fabrics, plastics, or other materials) when the cats were aged 6, 12, and 18 months were summarised.

### 2.6. Statistical Analysis of Pica toward Material Types and Cooccurrence between Timepoints

Cochran’s *Q* tests were run to determine if there were differences between the type of behaviour shown (pica and no pica) and the three time points for each of the four materials. To reduce the chances of a type I error being caused as a result of multiple testing, the Bonferroni correction was used. The critical *p*-value required was 0.0125. McNemar tests were used to provide post hoc analysis of variables included in Cochran’s *Q* tests where *p* < 0.0125.

A chi-square test was used to test for an association between pica reported in early life (Q2 and/or Q3) and subsequent pica reported in Q4.

### 2.7. Statistical Analysis of the Potential Risk Factors Associated with Chronic Pica

The statistical package IBM SPSS Statistics for Windows (Version 26) (IBM Corp: Armonk, NY, USA) was used for univariable and multivariable logistic regression analyses. In the univariable analysis, there was some variation in sample size for the potential explanatory variables due to some owners having not completed all questions within the four questionnaires.

Where variables had a category that contained no cases, one control was randomly selected using a random number generator (Research Randomizer—http://www.randomizer.org/ - accessed 30 June 2020) and recoded as a case so that the univariable model could be fitted to the data. After each alteration, the data were restored to the original format.

Variables found to have a univariable *p*-value of < 0.2 were included in the building of a multivariable model using the backward elimination technique. To facilitate comparison of models, cats with missing data for any of the eligible variables were excluded from the dataset. If two variables were found to be highly correlated (|r| > 0.9), one variable was excluded based on the creation of two models, each including one variable, and the model with the highest log-likelihood was selected. At the final stage of model building, all variables with *p*-value of < 0.05 were retained in the final model. The Hosmer and Lemeshow test was used to assess the fit of the model to the dataset.

## 3. Results

### 3.1. Prevalence and Cooccurrence of Pica within the Bristol Cats Study Cohort Reported in Questionnaires 2, 3 and 4

The number of cats recruited to the BCS was 2203, 64.6% (n = 1423) of their owners completed Q1–4 (inclusive). There were 889 cats that were excluded due to non-completion or partial completion of the pica question in Q2–4, leaving data from 534 cats eligible for descriptive analysis. For the risk factor analysis, a further 250 cats were excluded due to not meeting the outcome category criteria as pica was intermittently reported at just one or two of the time points (Q2–4) and thus could not be classified as chronic pica. This left 284 cats that were eligible for inclusion in the univariable analysis. To enable comparison of multivariable models, 113 cats were excluded because of missing data for variables that were to be included in the multivariable analysis. The resulting sample available for analysis consisted of 171 cats.

Table 2 shows the prevalence of owner-reported pica, and the Cochran’s *Q* tests results reveal highly significant associations between the age of the cat and the behaviours exhibited towards all four material types. Post hoc tests revealed that the prevalence of owner-reported pica significantly decreased between Q2 and Q3 (i.e., between approximate ages 6 months and 12 months) for all types of materials. In contrast, the prevalence of owner-reported pica was not significantly different between Q3 and Q4, although the prevalence of owner-reported pica for all four types of materials was significantly lower at Q4 when compared with Q2.

A chi-square test revealed a highly significant association between pica in early life (Q2 and/or Q3) and subsequent pica reported in Q4 (Table 3). Of the 165 cats that exhibited pica in Q4, 81.2% (n = 134) were also reported to show the behaviour in Q2 and/or Q3. Also, importantly, of the 280 cats that showed pica in early life, 52.1% (n = 146) did not show pica at Q4.

The cooccurrences of pica towards different material types are summarised in Table 4. In Q2, Q3 and Q4, 42.9% (229/534), 32.0% (171/534), and 30.9% (165/534) of cats were reported to express pica, respectively. At all three timepoints, it was most common for only one material type to be targeted as 47.2% (108/229), 55.0% (94/171), and 58.2% (96/165) of cats targeted only one material type in Q2, Q3, and Q4, respectively.

### 3.2. Early-Life Risk Factors for Pica

#### 3.2.1. Univariable Analysis

Of the 534 cats for which pica data from Q2–4 were available, 53.2% (n = 284) were eligible for inclusion in the risk factor analysis due to meeting the criteria for the two outcome categories. Of these 284 cats, 21.5% (n = 61) exhibited pica towards one or more material types at all three time points and 78.5% (n = 223) did not express pica at all three time points. Table S2 in Supplementary Materials summarises the univariable logistic regression. There were 27 variables with a *p* < 0.2 identified for inclusion in the multivariable model building process, however, seven variables were excluded due to being highly correlated. These variables were: single or multi-cat household, cat receives food treats, and frequency with which household members played with the cat per week. Breed and neuter status were not found to be significant at the univariable analysis.

#### 3.2.2. Multivariable Analysis

Three variables were retained in the final multivariable model (Table 5). Cats that lived in a rented home had increased odds of exhibiting chronic pica (reported in Q1), OR (95% CI) = 3.41 (1.45–8.03), compared to cats living in homes owned by their owners. Also, cats belonging to owners who had moved to a new house (reported in Q3) had increased odds of displaying chronic pica, OR (95% CI) = 13.95 (1.41–138.25) compared to cats whose owners had not reported moving house in Q3.

Cats living in a household without dogs (reported in Q1) had increased odds of being reported by their owners to exhibit chronic pica, OR (95% CI) = 4.86 (1.24–18.95) compared to cats living in households where dogs were present.

The final multivariable logistic regression model for the chronic pica was found to correctly classify 82.5% of cases; the Hosmer and Lemeshow test provided evidence that the model was a fair fit for the data (0.269).

## 4. Discussion

This study has presented descriptive data on the prevalence of pica towards non-nutritive items exhibited by cats at three data collection points (Q2, Q3 and Q4 when cats were aged 6.5–7 months, 12.5–13 months, and 18.5–19 months respectively). To the authors’ knowledge, this is the first study to examine the prevalence of pica within a longitudinal study of UK-owned pet cats. Most existing research in this field has explored pica within populations that were potentially subject to selection bias as the authors purposefully recruited cat breeds to their studies that were thought to be inclined to exhibit pica.

Bradshaw and others reported that onset most commonly occurred between 2–4 months of age, and also between 6–18 months of age [1]. The Cochran’s *Q* tests in this current study revealed highly significant associations between the cat age and pica exhibited towards all four material types. Post hoc McNemar tests revealed pica was significantly more likely to be reported in early life (Q2 than in Q3 and Q4). For all four of the material types, pica was most commonly reported at Q2 (6.5–7 months of age). Also, a chi square test revealed a highly significant association between pica in early life (Q2 and/or Q3) and subsequent pica reported in Q4. However, more than half (52.1%) of the 280 cats that showed pica in early life did not show pica at Q4. These statistical results suggest that pica declines in prevalence after initial onset, although some cats appear to retain the behaviour into adulthood.

This study investigated the cooccurrence of pica towards different material types. At all timepoints, it was most common for only one material type to be targeted, and the most commonly targeted material type was plastics. These findings are contradictory to those of Bradshaw and others who reported it was most common (34.2% of 152 cats) for three types of materials to be targeted [1]. Bradshaw and others reported a preference for fabrics as 93% of cats in their study targeted wool, 64%—cotton, 53%—synthetic fabrics, and only 22% targeted rubber or plastic materials [1]. However, in our study, and the study of Demontigny-Bédard and others [3], fabrics were not the preferred target item. Demontigny-Bédard and others reported that “shoelaces or threads” and “plastics” were the two most commonly ingested items, and plastics were the most chewed material type of the 73% of 100 cats that chewed objects [3]. There could be a number of explanations for the apparent preference for plastics observed in this current study. This could indicate a difference in material preference between populations of cats and/or availability of the material to the cats. Alternatively, the difference could have arisen because of the nature of the study: In this prospective study, owners were asked specifically to look for and report signs of chewing, and the evidence of chewing behaviours might be more noticeable on plastics than on fabric or other items. For example, teeth marks on a hard plastic item will probably be permanent, whereas chewing on fabric may not leave a visible mark unless a hole was made. Additionally, it is possible that the prevalence of pica towards “other materials” was under-reported, as no explicit examples of “other materials” were provided with the question and it was left to the participants to interpret.

The multivariable logistic regression analysis revealed that stability of the environment in which the cat lived appeared to influence the expression of chronic pica. Cats belonging to owners who had moved to a new house (reported in Q3 for the previous 6-month period when cats were aged 12.5–13 months) had increased odds of displaying chronic pica, than cats whose owners had not reported moving to a new house in Q3. Previous studies have suggested that stressful events could influence pica [1]. Novelty maybe be stressful [15,16] and a new environment could provide many stimuli that could induce stress. Why moving to a new home should have a larger impact on a cat aged approximately 12.5–13 months than a cat aged 6.5–7 months is unknown. However, it is speculated that an older cat may be more affected due to being more established in the original home than a younger cat. Further work would be useful to explore this association, particularly due to the large confidence interval and the variation in effect according to age of cat.

Cats with increased odds of expressing chronic pica were found to belong to owners who rented their home (reported in Q1), rather than owners who owned their home. A potential explanation for this is that owners may be more likely to react to their cat chewing items in a rented property compared to a property they owned. If the owner tries to distract the cat from expressing pica by interacting with it, this could have a reinforcing effect [17]. It is also possible that renting or owning a property could be a proxy for socio-economic factors or this could be confounding from other variables. This warrants further investigation.

Finally, the presence of dogs within the cats’ environment was found to influence the reported presence/absence of chronic pica. Cats living in households without dogs (reported in Q1—aged 6.5–7 months) had increased odds of exhibiting chronic pica, compared with cats living in households with dogs. This could suggest that either a familiar dog or dogs within a household have a protective effect on the expression of pica (for example, due to increased opportunity to show social behaviour and/or few periods of time without company), or factors within the environment prevent the cats from displaying pica, or pica being observed by owners (for example, avoidance of areas occupied by the dog (or dogs)). Without information on the relationship between the cat and dog (or dogs) in the household, this can only be speculated on and more research is required. It should be acknowledged that data on the presence of dogs within the cat’s household as reported in Q1 was analysed. Changes may have occurred within the household regarding dog ownership, so this finding should be interpreted with caution.

It should be acknowledged that owners were not asked how frequently their cats exhibited pica, as data on pica were collected as part of a questionnaire collecting information on many aspects of the cats’ lives at that timepoint. Therefore, the frequency of the behaviour shown by cats classified as chronically exhibiting pica will vary, and some cats that showed the behaviour may have done so infrequently and potentially not to the extent of clinical or behavioral concern. Also, it is possible that owners were subject to panel conditioning due to becoming more aware and/or looking for signs of pica following answering questions about the behaviour in the BCS questionnaires. However, the data presented here is a useful addition to existing research and can be used to direct future work into pica.

## 5. Conclusions

This study found pica was most commonly reported by owners in Q2 (6.5–7 months) and declined thereafter. This potentially indicates that pica is a kitten behaviour that is not necessarily continued with increasing age in all cats. Awareness of this finding might provide owners with reassurance should they be concerned by seeing their cat exhibiting pica when a kitten. Also, awareness of the factors associated with chronic pica reported in this study could help owners observing pica in young cats to potentially address the cat’s environment and the stability of that environment and reduce the odds of the behaviour becoming chronic. Other factors not explored in this study, such as how the owner responses to the cat exhibiting pica behaviour may be of great importance to subsequent behaviour, and this would be a valuable future area of research. This study moves forward understanding of the complexities of pica in cats and we hope provides direction for future research.

## Figures and Tables

**Table 1:** Variables assessed as potential risk factors for owner-reported chronic pica.

Variable	Description	Collapsed Categories
Sex (Q1)	Sex of the cat	Female
		Male
Breed (Q1)	Breed of the cat	Domestic shorthairs, domestic longhairs, and their crossbreeds
		Purebreds
Acquisition age (Q1)	Age of the cat when acquired by owners	<10 weeks
		≥10–20 weeks
		Since birth
Source of cat (Q1)	Where/how the owners obtained the cat	Accidentally or deliberately bred from an existing cat in the owners’ household
		From a pedigree breeder
		Rescue shelter/charity
		Stray/Feral/Found kitten/Kitten turned up at house
		All other sources
Neuter status	Variable derived from questions asking the age of the cat when neutered if neutered	Neutered between Q1–4 (approximately two—19 months)
		Not neutered by Q4
Indoor/outdoor access (Q2)	What indoor and outdoor access the cat was given	Inside only—not allowed out
		Access to outdoors
Indoor/outdoor access (Q3)	What indoor and outdoor access the cat was given	Inside only or restricted outdoor access via an enclosed run or on a lead
		Access to outdoors
Frequency with which household members played with the cat per week (Q2) (Q3)	Estimated frequency with which household members played with the cat in a week	Most days
		Quite often (1–2 times/week)/Not very often (1–3 times/month)/Never
Change in “frequency with which household members played with the cat per week” (Q2 and Q3)	This variable was derived from the responses given in **both** Q2 and Q3	Consistently most days
		Consistently quite often (1–2 times/week)/Not very often (1–3 times/month)
		Change in frequency over time
Ill or injured (Q1)	Illness or injury reported that may or may not have required a visit to a vet	No
		Yes
Ill or injured (Q2)	Illness or injury reported that required a visit to a vet	No
		Yes
Owner’s opinion on cat’s appetite (Q2) (Q3)	Owner’s opinion on their cat’s appetite	Very good
		Fairly good/not very good/not at all good
Change in “owner’s opinion on cat’s appetite” (Q2 and Q3)	This variable was derived from the responses given in **both** Q2 and Q3	Consistently very good
		Consistently fairly good/not very good
		Change in appetite reported
Cat receives food treats (Q1) (Q2) (Q3)	Owners were asked if they gave food treats to their cat	Every day/several times a week/twice a week or less often
		Never
Change in “cat receives food treats” (Q1 and Q2) (Q2 and Q3) (Q1, Q2 and Q3)	This variable was derived from the responses given in Q1, Q2 and Q3	Consistently yes
		Consistently never
		Change
Single or multi-cat household (Q1) (Q2) (Q3)	This variable was derived from owners who reported cats had joined or left the household	Single cat household
		Multi-cat household
Change in “single or multi-cat household” (Q1 and Q2) (Q2 and Q3) (Q1, Q2 and Q3)	This variable was derived from the responses given in Q1, Q2 and Q3	Consistently a single cat household
		Consistently a multi-cat household
		Change in the number of cats
Presence of a dog(s) in household (Q1)	Presence of a dog(s) in the household	No
		Yes
Number of adults in household (Q1)	Number of adults in household	1 or 2 adults
		3 or more adults
Presence of children in household (Q1)	Presence of children in the household	No
		Yes
Housing tenure	Housing tenure	Own (with or without mortgage)
		Rent house, or house comes with employment
Moved to a new house (Q2) (Q3)	Owner moved to a new house since the last questionnaire	No
		Yes
Change in “moved to a new house” (Q2 and Q3)	This variable was derived from the responses given in **both** Q2 and Q3	No
		Yes
Annual household income (Q1)	Annual household income	<£15,000
		≥£15,000
Highest level of education (Q1)	Highest level of education that any member of household has achieved	No qualifications/GCSEs/O’ levels
		A’ levels
		HND/Degree Post-graduate/ Professional qualifications

KEY—Questionnaire numbers in brackets indicate when data for those variables were collected. For example, “Moved to a new house (Q2) (Q3)” indicates two separate variables as data were in Q2 and Q3. Where two questionnaire numbers are in the same set of brackets, two questionnaires’ responses were combined. Questionnaires were issued between May 2010 and April 2015 when their cat reached specific ages. Questionnaire 1 (Q1): 2–4 months, Questionnaire 2 (Q2): 6.5–7 months, Questionnaire 3 (Q3): 12.5–13 months and Questionnaire 4 (Q4): 18.5–19 months.

**Table 2:** Owner-reported pica prevalence and the results of Cochran’s *Q* tests and post hoc McNemar tests for differences between behaviour type and the three time points for each material (n = 534).

Type of Material	Behaviour Reported	Number of Cats Reported at Time Point (%)			*p*-Value ^d^	Post Hoc Tests	
		Q2 ^a^ n = 534	Q3 ^b^ n = 534	Q4 ^c^ n = 534		Timepoints	*p*-Value ^e^
**Woollen fabrics**	Chews with or without ingesting	66 (12.4)	44 (8.2)	35 (6.6)	<0.001	Q2-Q3 Q3-Q4 Q2-Q4	0.006 0.243 <0.001
	No pica behaviour	468 (87.6)	490 (91.8)	499 (93.4)			
**Other fabrics**	Chews with or without ingesting	84 (15.7)	44 (8.2)	46 (8.6)	<0.001	Q2-Q3 Q3-Q4 Q2-Q4	<0.001 0.890 <0.001
	No pica behaviour	450 (84.3)	490 (91.8)	488 (91.4)			
**Plastics**	Chews with or without ingesting	120 (22.5)	91 (17.0)	94 (17.6)	0.005	Q2-Q3 Q3-Q4 Q2-Q4	0.005 0.828 0.015
	No pica behaviour	414 (77.5)	443 (83.0)	440 (82.4)			
**Other materials**	Chews with or without ingesting	167 (31.3)	109 (20.4)	97 (18.2)	<0.001	Q2-Q3 Q3-Q4 Q2-Q4	<0.001 0.299 <0.001
	No pica behaviour	367 (68.7)	425 (79.6)	437 (81.8)			

Using Bonferroni’s correction the critical *p*-value required was 0.0125. ^a^ Questionnaire 2 was completed when cats were aged 6.5–7 months. ^b^ Questionnaire 3 was completed for cats aged 12.5–13 months. ^c^ Questionnaire 4 was completed when cats were aged 18.5–19 months. ^d^ Cochran’s *Q* test ^e^ McNemar test.

**Table 3:** Results of a chi square test for association between pica reported in early timepoints (Questionnaire 2 and/or 3) and Questionnaire 4 (n = 534).

	Pica Reported in Q4 ^c^			
Pica Reported in Q2 ^a^ and/or Q3 ^b^	Yes N (%)	No N (%)	*X* ^2^	*p*-Value
Yes	134 (81.2)	146 (39.6)		
No	31 (18.8)	223 (60.4)	79.286	<0.001

^a^ Questionnaire 2 was completed when cats were aged 6.5–7 months. ^b^ Questionnaire 3 was completed for cats aged 12.5–13 months. ^c^ Questionnaire 4 was completed when cats were aged 18.5–19 months.

**Table 4:** Co-occurrence of pica exhibited by 229, 171, and 165 cats who targeted one or more material types as reported in Questionnaire 2 (when cats were aged 6.5–7 months), Questionnaire 3 (when cats were aged 12.5–13 months) and Questionnaire 4 (when cats were aged 18.5–19 months).

	Number of Cats Reported Expressing Pica (%)														
	Questionnaire 2					Questionnaire 3					Questionnaire 4				
Number and Type of Material Targeted	Woollen Fabrics	Other Fabrics	Plastics	Other Materials	Total (%)	Woollen Fabrics	Other Fabrics	Plastics	Other Materials	Total (%)	Woollen Fabrics	Other Fabrics	Plastics	Other Materials	Total (%)
**One Material Targeted**															
	9 (8.3)	13 (12.0)	29 (26.9)	57 (52.8)	108 (47.2)	11 (11.7)	6 (6.4)	30 (31.9)	47 (50.0)	94 (55.0)	8 (8.3)	10 (10.4)	40 (41.7)	38 (39.6)	96 (58.2)
**Two Materials Targeted**															
Woollen fabrics	-	-	-	-		-	-	-	-		-	-	-	-	
Other fabrics	5 (7.9)	-	-	-		10 (20.0)	-	-	-		3 (7.3)	-	-	-	
Plastics	4 (6.4)	1 (1.6)	-	-		1 (2.0)	0 (0.0)	-	-		2 (4.9)	1 (2.4)	-	-	
Other materials	5 (7.9)	11 (17.5)	37 (58.7)	-	63 (27.5)	1 (2.0)	3 (6.0)	35 (70.0)	-	50 (29.2)	3 (7.3)	6 (14.6)	26 (63.4)	-	41 (24.8)
**Three Materials Targeted**															
Other fabrics + Plastics	1 (3.5)	-	-	-		4 (28.6)	-	-	-		4 (22.2)	-	-	-	
Other fabrics + Other materials	9 (31.0)	-	-	-		2 (14.3)	-	-	-		3 (16.7)	-	-	-	
Plastics + Other materials	4 (13.8)	15 (51.7)	-	-	29 (12.7)	2 (14.3)	6 (42.9)	-	-	14 (8.2)	2 (11.1)	9 (50.0)	-	-	18 (10.9)
**Four Materials Targeted**															
Other fabrics + Plastics + Other materials	29 (100.0)	-	-	-	29 (12.7)	13 (100.0)	-	-	-	13 (7.6)	10 (100.0)	-	-	-	10 (6.1)
**Total**	66 (28.8)	40 (17.5)	66 (28.8)	57 (24.9)	229 (100.0)	44 (25.7)	15 (8.8)	65 (38.0)	47 (27.5)	171 (100.0)	35 (21.2)	26 (15.8)	66 (40.0)	38 (23.0)	165 (100.0)

**Table 5:** Multivariable logistic regression model summarising explanatory variables that were associated with chronic pica.

Variable	Categories	Controls n (%)	Cases n (%)	OR (95% CI)	*p*-Value
Moved to a new house *Reported in Q3* ^b^	No	135 (82.3)	29 (17.7)	1.00	
	Yes	1 (14.3)	6 (85.7)	13.95 (1.41–138.25)	0.024
Housing tenure *Reported in Q1*	Own (with or without mortgage)	102 (87.2)	15 (12.8)	1.00	
	Rent home, or house comes with employment	34 (63.0)	20 (37.0)	3.41 (1.45–8.03)	0.005
Presence of a dog(s) in household *Reported in Q1* ^a^	Yes	35 (92.1)	3 (7.9)	1.00	
	No	101 (75.9)	32 (24.1)	4.86 (1.24–18.95)	0.023

^a^ Questionnaire 1 was completed when cats were aged 2–4 months. ^b^ Questionnaire 3 was completed for cats aged 12.5–13 months.

## Data Availability

Data available from authors on request.

## Supplementary Materials

The following are available online at https://www.mdpi.com/article/10.3390/ani11041101/s1, Table S1: Questions regarding pica, Table S2: Univariable logistic regression analysis of potential variables for owner-reported chronic pica.:
