# Supplementary material for: Owner-Reported Pica in Domestic Cats Enrolled onto a Birth Cohort Study
Source: Animals (Basel). 2021 Apr 12;11(4):1101. doi: 10.3390/ani11041101 (PMC8070519; doi:10.3390/ani11041101)
Supplement: Supplementary file 1 [file animals-11-01101-s001.pdf]

animals MDPI

Supplementary Materials

Table S1: Questions regarding pica

In Questionnaires 2, 3 and 4, owners were asked:

Please indicate which, if any, of these materials your cat currently chews and/or eats and which your cat used to chew and/or eat.					
	Currently chews but does not eat	Currently chews and eats	Used to chew or eat but no longer does so	Has never chewed or eaten	Don't know
Woollen fabrics					
Other fabrics					
Plastics					
Other Materials					

Table S2: Univariable logistic regression analysis of potential variables for owner-reported chronic pica.

Variable	Categories	Controls n (%)	Cases n (%)	OR (95% CI)	P-Value
**Sex**	Female	123 (82.0)	27 (18.0)	1.00	
	Male	100 (74.6)	34 (25.4)	1.55 (0.88-2.74)	0.132
**Breed**	Domestic shorthairs, domestic longhairs, and their crossbreeds	162 (79.8)	41 (20.2)	1.00	
	Purebreds	59 (74.7)	20 (25.3)	1.34 (0.73-2.47)	0.349
**Acquisition age**	<10 weeks	126 (77.3)	37 (22.7)	1.00	0.074
	≥10 weeks	66 (74.2)	23 (25.8)	1.19 (0.65-2.16)	0.576
	Since birth	31 (96.9)	1 (3.1)	0.11 (0.02-0.83)	0.033
**Source of cat**	Accidentally or deliberately bred from an existing cat in the owners' household	25 (89.3)	3 (10.7)	1.00	0.205
	From a pedigree breeder	45 (70.3)	19 (29.7)	3.52 (0.95-13.07)	0.060
	Rescue shelter/charity	79 (76.7)	24 (23.3)	2.53 (0.70-9.12)	0.155
	Stray/Feral/Found kittens/Kitten turned up at house	51 (82.3)	11 (17.7)	1.80 (0.46-7.03)	0.399
	All other sources	20 (87.0)	3 (13.0)	1.25 (0.23-6.88)	0.798
**Neuter status**	Neutered prior to Q4 [c]	205 (77.7)	59 (22.3)	1.00	
	Not neutered by Q4	18 (90.0)	2 (10.0)	0.39 (0.09-1.71)	0.210
**Indoor/outdoor access** ***Reported in Q2*** [a]	Inside only – not allowed out	68 (69.1)	30 (30.9)	1.00	
	Access to outdoors	153 (83.2)	31 (16.8)	0.46 (0.26-0.82)	0.008
Reported in Q3 [b]	Inside only or restricted outdoor access via an enclosed run or on a lead	97 (82.2)	21 (17.8)	1.00	
	Access to outdoors	100 (73.5)	36 (26.5)	1.66 (0.91-3.05)	0.100
**Frequency with which household members played with the cat per week. *Reported in Q2***	Most days	202 (77.1)	60 (22.9)	1.00	
	Quite often/Not very often/Never	20 (95.2)	1 (4.8)	0.17 (0.02-1.28)	0.085
Reported in Q3	Most days	157 (73.0)	58 (27.0)	1.00	
	Quite often/Not very often/Never	64 (95.5)	3 (4.5)	0.13 (0.04-0.42)	0.001

*Reported in Q2 and Q3 **	Consistently most days	149 (72.3)	57 (27.7)	1.00	0.002
	Consistently quite often/not very often	11 (91.7)	1 (8.3)	0.24 (0.03-1.88)	0.174
	Change in frequency over time	60 (95.2)	3 (4.8)	0.13 (0.04-0.43)	0.001
**Ill or injured.** *Reported in Q1* **– may or may not have required visit to a vet**	No	178 (79.1)	47 (20.9)	1.00	
	Yes	42 (77.8)	12 (22.2)	1.08 (0.53-2.22)	0.829
**Ill or injured.** *Reported in Q2* **– required a visit to a vet**	No	199 (79.9)	50 (20.1)	1.00	
	Yes	24 (68.6)	11 (31.4)	1.82 (0.84-3.97)	0.130
**Owner's opinion on cat's appetite.** *Reported in Q2*	Very good	188 (79.0)	50 (21.0)	1.00	
	Fairly good/not very good/not at all good	31 (75.6)	10 (24.4)	1.21 (0.56-2.64)	0.627
Reported in Q3	Very good	153 (75.7)	49 (24.3)	1.00	
	Fairly good/not very good/not at all good	63 (84.0)	12 (16.0)	0.60 (0.30-1.19)	0.143
Reported in Q2 and Q3	Consistently very good	142 (76.3)	44 (23.7)	1.00	0.484
	Consistently fairly good/not very good	19 (76.0)	6 (24.0)	1.02 (0.38-2.71)	0.970
	Change in appetite reported	51 (83.6)	10 (16.4)	0.63 (0.30-1.35)	0.236
**Cat receives food treats** *Reported in Q1*	Yes	143 (76.1)	45 (23.9)	1.00	
	Never	78 (83.9)	15 (16.1)	0.61 (0.32-1.17)	0.135
Reported in Q2	Yes	166 (76.5)	51 (23.5)	1.00	
	Never	55 (84.6)	10 (15.4)	0.59 (0.28-1.24)	0.167
Reported in Q3	Yes	169 (76.1)	53 (23.9)	1.00	
	Never	50 (86.2)	8 (13.8)	0.51 (0.23-1.14)	0.102
*Reported in Q1 and Q2 **	Consistently yes	124 (76.5)	38 (23.5)	1.00	0.060
	Consistently never	36 (94.7)	2 (5.3)	0.18 (0.04-0.79)	0.023
	Change	59 (74.7)	20 (25.3)	1.11 (0.59-2.06)	0.751
Reported in Q2 and Q3	Consistently yes	150 (75.4)	49 (24.6)	1.00	0.223
	Consistently never	38 (86.4)	6 (13.6)	0.48 (0.19-1.21)	0.121
	Change	29 (82.9)	6 (17.1)	0.63 (0.25-1.62)	0.339
*Reported in Q1, Q2 and Q3 **	Consistently yes	111 (75.0)	37 (25.0)	1.00	0.109
	Consistently never	26 (96.3)	1 (3.7)	0.12 (0.02-0.88)	0.037
	Change	78 (78.0)	22 (22.0)	0.85 (0.46-1.55)	0.586
**Single or multi-cat household.** *Reported in Q1*	Single cat household	36 (67.9)	17 (32.1)	1.00	
	Multi-cat household	186 (81.6)	42 (18.4)	0.48 (0.25-0.93)	0.030
*Reported in Q2 **	Single cat household	31 (66.0)	16 (34.0)	1.00	
	Multi-cat household	190 (81.2)	44 (18.8)	0.45 (0.23-0.90)	0.022
*Reported in Q3 **	Single cat household	27 (64.3)	15 (35.7)	1.00	
	Multi-cat household	194 (81.2)	45 (18.8)	0.42 (0.21-0.85)	0.016
*Reported in Q1 and Q2 **	Consistently a single cat household	29 (65.9)	15 (34.1)	1.00	0.069
	Consistently a multi-cat household	183 (81.7)	41 (18.3)	0.43 (0.21-0.88)	0.021
	Change in the number of cats	8 (80.0)	2 (20.0)	0.48 (0.09-2.57)	0.394
Reported in Q2 and Q3	Consistently a single cat household	27 (64.3)	15 (35.7)	1.00	0.054

	Consistently a multi-cat household	190 (81.2)	44 (18.8)	0.42 (0.21-0.85)	0.016
	Change in the number of cats	4 (80.0)	1 (20.0)	0.45 (0.05-4.40)	0.493
*Reported in Q1, Q2 and Q3 **	Consistently a single cat household	27 (65.9)	14 (34.1)	1.00	0.077
	Consistently a multi-cat household	183 (81.7)	41 (18.3)	0.43 (0.21-0.90)	0.024
	Change in the number of cats	10 (76.9)	3 (23.1)	0.58 (0.14-2.45)	0.457
**Presence of a dog(s) in household** *Reported in Q1*	No	154 (74.4)	53 (25.6)	1.00	
	Yes	61 (89.7)	7 (10.3)	0.33 (0.14-0.77)	0.011
**Number of adults in household** *Reported in Q1*	1 or 2 adults	156 (76.5)	48 (23.5)	1.00	
	3 or more adults	57 (89.1)	7 (10.9)	0.40 (0.17-0.93)	0.034
**Presence of children in household** *Reported in Q1*	No	162 (6.1)	51 (23.9)	1.00	
	Yes	58 (87.9)	8 (12.1)	0.44 (0.20-0.98)	0.044
**Housing tenure** *Reported in Q1*	Own (with or without mortgage)	161 (83.0)	33 (17.0)	1.00	
	Rent home, or house comes with employment	61 (69.3)	27 (30.7)	2.16 (1.20-3.89)	0.010
**Moved to a new house** *Reported in Q2*	No	212 (79.1)	56 (20.9)	1.00	
	Yes	11 (68.8)	5 (31.3)	1.72 (0.57-5.16)	0.332
Reported in Q3	No	207 (79.9)	52 (20.1)	1.00	
	Yes	9 (52.9)	8 (47.1)	3.54 (1.30-9.62)	0.013
Reported in Q2 and Q3	No	196 (80.3)	48 (19.7)	1.00	
	Yes	20 (62.5)	12 (37.5)	2.45 (1.12-5.36)	0.025
**Annual household income** *Reported in Q1*	<£15,000	42 (82.4)	9 (17.6)	1.00	
	≥£15,000	164 (78.1)	46 (21.9)	1.31 (0.59-2.89)	0.505
**Highest level of education** *Reported in Q1*	No qualifications/GCSEs/O' levels	35 (89.7)	4 (10.3)	1.00	0.048
	A Levels	28 (66.7)	14 (33.3)	4.38 (1.30-14.78)	0.017
	HND or degree/Post-graduate degree/Professional qualifications	150 (78.9)	40 (21.1)	2.33 (0.78-6.95)	0.128

[a] Questionnaire 2 was completed when cats were aged 6.5-7 months. [b] Questionnaire 3 was completed for cats aged 12.5-13 months. [c] Questionnaire 4 was completed when cats were aged 18.5-19 months. * Variables that were excluded from the multivariable model building process due to have missing data for more than 150 cats.
